# Tree Circumference Dynamics in Four Forests Characterized Using Automated Dendrometer Bands

**DOI:** 10.1371/journal.pone.0169020

**Published:** 2016-12-28

**Authors:** Valentine Herrmann, Sean M. McMahon, Matteo Detto, James A. Lutz, Stuart J. Davies, Chia-Hao Chang-Yang, Kristina J. Anderson-Teixeira

**Affiliations:** 1 Conservation Ecology Center, Smithsonian Conservation Biology Institute, National Zoological Park, Front Royal, VA, United States of America; 2 Smithsonian Environmental Research Center, Edgewater, Maryland, United States of America; 3 Center for Tropical Forest Science-Forest Global Earth Observatory, Smithsonian Tropical Research Institute, Panamá, Republic of Panamá; 4 Department of Ecology and Evolutionary Biology, Princeton University, Princeton, NJ, United States of America; 5 Wildland Resources Department, Utah State University, Logan, Utah, United States of America; 6 Department of Botany, National Museum of Natural History, Washington, DC, United States of America; 7 Department of Natural Resources and Environmental Studies, National Dong Hwa University, Hualien,Taiwan; Chinese Academy of Forestry, CHINA

## Abstract

Stem diameter is one of the most commonly measured attributes of trees, forming the foundation of forest censuses and monitoring. Changes in tree stem circumference include both irreversible woody stem growth and reversible circumference changes related to water status, yet these fine-scale dynamics are rarely leveraged to understand forest ecophysiology and typically ignored in plot- or stand-scale estimates of tree growth and forest productivity. Here, we deployed automated dendrometer bands on 12–40 trees at four different forested sites—two temperate broadleaf deciduous, one temperate conifer, and one tropical broadleaf semi-deciduous—to understand how tree circumference varies on time scales of hours to months, how these dynamics relate to environmental conditions, and whether the structure of these variations might introduce substantive error into estimates of woody growth. Diurnal stem circumference dynamics measured over the bark commonly—but not consistently—exhibited daytime shrinkage attributable to transpiration-driven changes in stem water storage. The amplitude of this shrinkage was significantly correlated with climatic variables (daily temperature range, vapor pressure deficit, and radiation), sap flow and evapotranspiration. Diurnal variations were typically <0.5 mm circumference in amplitude and unlikely to be of concern to most studies of tree growth. Over time scales of multiple days, the bands captured circumference increases in response to rain events, likely driven by combinations of increased stem water storage and bark hydration. Particularly at the tropical site, these rain responses could be quite substantial, ranging up to 1.5 mm circumference expansion within 48 hours following a rain event. We conclude that over-bark measurements of stem circumference change sometimes correlate with but have limited potential for directly estimating daily transpiration, but that they can be valuable on time scales of days to weeks for characterizing changes in stem growth and hydration.

## Introduction

Stem diameter is a commonly measured attribute of trees that is foundational to forest censuses and monitoring [1–4]. Tree stem diameter is tightly coupled to total tree mass [5,6], and diameter changes are routinely used to estimate woody growth rates and their seasonal patterns [7–9]. Changes in the rates of tree growth are also predictors of tree mortality [10] and competitive interactions [11]. Moreover, because tree stems shrink and swell in relation to their hydraulic status, high-resolution measurements of diameter changes can be used to infer rates of water use and hydraulic stress [12–17]. Scaled to the stand level, tree diameter changes are used in conjunction with allometric equations for estimating aboveground woody productivity [18], and are potentially useful for understanding hydraulic functions such as transpiration [19]. Therefore, the dynamics of tree diameter change at time scales ranging from minutes to years provide an important basis for characterizing multiple aspects of tree performance and forest-climate interactions.

Tree physiological studies have characterized how changes in tree stem diameter—typically measured using high-resolution point dendrometers installed under the outer bark—are shaped by both reversible circumference changes related to stem water status and irreversible stem growth. On a daily time scale, tree stems shrink and swell in relation to their water status [20]. The most commonly observed pattern is that trees attain their maximum circumference just before dawn, at which point stomata open and the trees start losing water to the atmosphere more rapidly than they are taking it up from the soil [20,21]. Changes in xylem water tension [16,22] and water storage in the bark and phloem [13,23] cause the tree stems to shrink, reaching a minimum in the afternoon and then swelling to a new maximum before the next dawn. Half-hourly variation in tree diameter often correlates with sap flow rate [16]. Moreover, because the amplitude of daily shrinkage is a function of water loss through the leaves and the water uptake by roots, daily shrinkage often correlates with evaporative demand, water supply or other that influence tree water use [14,24–26]. On the time scale of days to years, tree stems undergo irreversible growth as newly formed cells expand, and this signal combines with changes in stem water potential to shape dynamics of stem diameter change [27–29]. On this time scale, reversible changes can be driven by xylem water storage or bark hydration [30–32]. Stem shrinkage during dry spells has been commonly observed in both tropical [18] and extra-tropical [33] forests.

Despite the known importance of hydraulic-driven dynamics in tree stem diameter, hydraulic influences tend not to be characterized in plot- or stand-scale ecological studies—primarily because it is not currently feasible to monitor high-frequency changes in stem diameter on tens to hundreds of trees. One option for characterizing stem circumference dynamics over short time scales on the stand level is to use automated dendrometer bands (ADBs), which are often cheaper and easier to deploy than point dendrometers but differ in that they must be installed over the bark. If ADBs can detect daily fluctuations in circumference driven by changes in stem water content, they could serve as a tool to investigate tree hydraulic functions, such as transpiration and response to water stress [14,15,33]. Moreover, on the time scale of days to years, ADBs could serve as a tool to understand changes in stem hydration and provide fine-scale, unbiased estimates of stem growth and forest productivity.

In order to characterize tree circumference dynamics on time scales of hours to months in different types of forest, we deployed dozens of ADBs in two temperate broadleaf deciduous forests, one temperate conifer forest, and one tropical broadleaf semi-deciduous forest. We analyze the phase of the diurnal signal, assessing the extent to which the over-bark measurements of ADBs capture the pattern of daytime shrinkage and nighttime swelling that has been commonly observed using point dendrometers installed under the outer bark. We further examine these daily patterns in relation to meteorological variables and tree transpiration rates, testing whether daily shrinkage is greater when trees are more actively transpiring. In addition, we characterize rain responses, testing whether rain events trigger more rapid stem expansion.

## Methods

### Study sites

**Fig 1:**
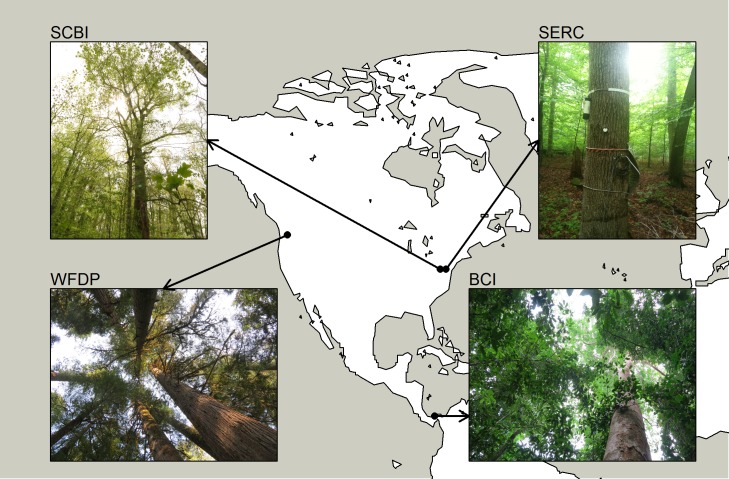
Study Locations.

This study was conducted at four long-term forest dynamics plots that are part of the Center for Tropical Forest Science-Forest Global Earth Observatory (CTFS-ForestGEO; forestgeo.si.edu; [3]; Fig 1). These included temperate deciduous forests at the Smithsonian Conservation Biology Institute (SCBI; Front Royal, VA, USA) and Smithsonian Environmental Research Center (SERC; Edgewater, MD, USA), an evergreen conifer forest in the T. T. Munger Experimental Forest (the Wind River Forest Dynamics Plot, WFDP; Wind River, WA, USA), and a semi-deciduous broadleaf forest at Barro Colorado Island (BCI; Panama). Site details are provided in Table 1. Köppen-Geiger climate zones were obtained from [3,34].

**Table 1:** Study sites details.

Site	Geographic coordinates	Elevation (m.a.s.l.)	Mean annual temperature (°C)	Mean annual precipitation (mm)	Köppen-Geiger climate zone^a^	Dominant vegetation	Refs.
Smithsonian Conservation Biology Institute (SCBI)	38.8935 N, 78.1454 W	273–338	12.9	1,001	Cfa	Broadleaf deciduous *(Liriodendron tulipifera*, *Quercus spp*., *Carya spp*.,*)*	[35,36]
Smithsonian Environmental Research Center (SERC)	38.8891 N, 76.5594 W	6–10	13.2	1,068	Cfa	Broadleaf deciduous *(Liriodendron tulipifera*, *Quercus spp*., *Fagus grandifolia*, *Carya spp*.*)*	[7]
Wind River (WFDP)	45.8197 N, 121.9558 W	352–385	9.2	2,495	Csb	Needleleaf evergreen (*Pseudotsuga menziesii*, *Tsuga heterophylla*)	[11,37]
Barro Colorado Island (BCI)	9.1543 N, 79.8461 W	120–160	27.1	2,551	Am	Broadleaf evergreen, broadleaf drought deciduous (diverse)	[38–40]

^a^ Am- tropical monsoon; Cfa- warm temperate with year-round precipitation; Csb- temperate dry summer.

### Ethics statement

The SCBI, SERC, and BCI sites are on land administered by the Smithsonian Institution. Permission for work at SCBI was obtained from the SCBI Land Use Committee. For BCI, this work was conducted as part of MDD’s project “Monitoring carbon fluxes in tropical forest”, which was approved by the Smithsonian Tropical Research Institute Scientific Permit Office. At SERC, special permission was not required for a project involving a SERC staff member (SMM). The WFDP operates under a permit issued by the Research Natural Area Program of the Pacific Northwest Research Station, USDA Forest Service, currently valid through 12/31/2020. No protected species were sampled.

### Measurements

ADBs were deployed in 2014 at SCBI (n = 12 bands) and in 2015 in the other sites (n = 33–40 bands), as detailed in Table 2 and in S1 Appendix. Attempted deployment of bands at SCBI in May 2015 was canceled because PVC tubes containing data loggers were consistently pulled off the trees by bears within few days. Bands were located either within clusters of trees monitored for sap flow (SCBI, SERC) or within the fetch of an eddy-covariance tower (WFDP, BCI). Trees were selected to span the range of sizes ≥15 cm diameter present at the site and to represent 2–5 species or functional groups. At SCBI, SERC, and WFDP, 1–3 dead trees were instrumented to characterize changes in circumference in the absence of growth or active water transport. At SERC, 11 trees were located in an irrigated plot created during a separate project to explore water supplementation on tree growth.

**Table 2:** Measurement details by site.

Site	Taxa instrumented	DBH range (cm)	band installation date(s)	dates analyzed	n trees with useable data(n instrumented)	% useable data: mean (range)^a^	ADB location	Weather station location
SCBI	*Fagus grandifolia (5)*, *Liriodendron tulipifera (5)*, dead (2).	14.9–105.9	May 05, 2014	June 19 –August 19, 2014	12 (12)	89.12 (56.43–100.00)	two 700-m^2^ clusters instrumented with sap flow probes within CTFS-ForestGEO plot	Clearing just outside CTFS-ForestGEO plot
SERC	*Acer rubra (1)*, *Carya* spp. *(7)*, *Fagus granduifolia (11)*, *Liquidambar styraciflua (3)*, *Lirodendron tulipifera (7) Quercus* spp. *(5)*, dead (1).	16.2–111.6	June 15, 2015	June 19 –August 19, 2015	28 (35)	95.2 (78.09–100.00)	two 700-m^2^ clusters instrumented with sap flow probes outside CTFS-ForestGEO plot, one of which was watered.	Above-canopy tower outside CTFS-ForestGEO plot (~40m).
WFDP	*Abies amabilis (5)*, *Pseudotsuga menziesii (9)*, *Thuja plicata (9)*, *Tsuga heterophylla (9)*, dead (3).	14.4–180.7	April 20–21, 2015	April 25- June 25, 2015	37 (40)	98.68 (72.09–100.00)	200 x 200 m area centered around eddy-flux tower within CTFS-ForestGEO plot	Above-canopy tower within CTFS-ForestGEO plot
BCI	13 species; see S1 Appendix.	21–137.5	June 22–26, 2015	July 1- Sept. 1, 2015	31 (34)	89.09 (54.71–100.00)	200 x 300 m area centered around eddy-flux tower outside CTFS-ForestGEO plot	Above-canopy tower outside CTFS-ForestGEO plot

^a^Only for trees with usable data.

The ADBs (TreeHugger, Global Change Solutions, LLC; Urbana, IL, USA), consisted of a linear potentiometer and an adjustable stylus mounted on a stainless steel band (0.015 × 1.9 cm; SAE type 301 stainless steel) tensioned with stainless steel springs. Changes in circumference were recorded as a change in resistance as the stylus moved across the potentiometer. Band temperature was recorded by a thermistor mounted on the potentiometer. Dedicated data loggers associated with each unit were contained in PVC tubes that hung on each tree. Loggers recorded to MicroSD card and were powered by two AA batteries. Units were manufactured a few months prior to their deployment at each site. Minor design differences between installations included improvements to battery life and waterproofing of the potentiometer and should not affect results presented here.

Bands were installed at ~1.3 m height, adjusted to avoid placement at the exact height of CTFS-ForestGEO census measurements and stem abnormalities such as branches, knots, or buttresses. At BCI, bands were installed at heights of up to 4 m to avoid buttresses. Prior to installing bands, stem surfaces were prepared to ensure a snug fit for the band; for instance, moss, epiphytic roots or lianas were removed or avoided (e.g., by placing band under the liana), loose outer bark was lightly smoothed with a rasp, thorns were knocked off or filed flat (e.g., *Hura crepitans* at BCI), and tracks for the band were cut into bark ridges on some large trees with very thick bark (e.g., *Pseudotsuga menziesii* at WFDP). Bands were fit as snugly as possible, with the measurement window located over a smooth and regular portion of the stem. Diameter at band height was recorded, and the initial width of a manual measurement window (defined by physical markings on the band) measured to the nearest 0.01 mm using high-precision digital calipers (Mitutoyo 500-196-20). PVC tubes containing data loggers were secured 10–30 cm above or below the band, hung with plastic pipe hanger wrapped around the tree or, for conifers, secured with a nail. Data were recorded at 15-minute intervals. Trees were visited to check instrument status, download data, and make manual measurements at intervals ranging from weeks (SCBI, SERC) to months (BCI, WFDP). Some data were lost due to SD card failures, battery failures, or physical damage to the instruments (e.g., tearing of potentiometer connection, corrosion caused by moisture, damage by wildlife; see S1 and S2 Appendices). The temperature response of the system was tested with bands tensioned around a cylindrical glass object with a lower thermal expansion coefficient, less friction, and lower thermal inertia than a tree, and based on this we concluded that it would not be appropriate to apply a thermal correction to the dendrometer band readings (see S4 Appendix).

In addition to the ADBs, a variety of meteorological variables and metrics of transpiration were recorded at each site. Weather stations at each site (Table 2) measured air temperature (*T*_*air*_; °C), relative humidity (*RH*; %), precipitation (*PPT*; mm), and solar radiation (*Rad*; W m^-2^). Vapor pressure deficit (*VPD*; kPa) was calculated from *T*_*air*_ and *RH*. Ecosystem-atmosphere exchange of H_2_O (evapotranspiration, *ET*; g mol^-1^) and sap flux density (*SF*, m h^-1^). *ET* was measured using eddy covariance systems at WFDP (Bible & Wharton, 1998–2015—AmeriFlux US-Wrc Wind River Crane Site, DOI: 10.17190/AMF/1246114) and BCI (Detto, unpublished data). *SF* was measured at SCBI (n = 28) and SERC (n = 30) using Granier-style probes [41] on trees within the same clusters as the dendrometer bands. Methods used for sap flow data processing at SCBI are described in Ref. [36], data were processed in a similar way at SERC. For both sites, ensemble *SF* time series were created for each genus using the median value of records for the genus. All measurements were collected at time interval of 1 to 30 minutes. To produce time series aligning with ADB time stamps, we resampled 15-minute intervals by linear interpolation (for lower frequency data) or aggregation (for higher frequency data).

### Analyses

We selected a two-month analysis period starting ≥4 days after band installation (Table 2) for each site. This period encompassed early- to mid- growing season at the three temperate sites or, at BCI, the dry season—wet season transition, during a strong El Niño event. All data analyses were conducted in R version 3.2.1 [42].

Data were first screened to remove bad values. Electronic measurements of change in sensor position were paired to manual measurements of the change in the measurement window. When the rate of change of the sensor position was off by more than 4mm compared to the rate of change of the manual measurement window, all sensor data recorded between the 2 manual measurements that showed discrepancy were excluded. This step was not performed for BCI, which did not have repeated manual measurements. Second, we removed all data from any day that had a sensor position change of more than 0.5 mm (or 1 mm for BCI) within an hour. Finally, we manually screened the remaining data, removing unrealistic isolated records that were not automatically discarded when sensors stabilized after sudden jumps. Trees with <50% of data remaining following this screening procedure were completely excluded from further analysis (see S1 and S2 Appendices for details about failure rates).

**Fig 2:**
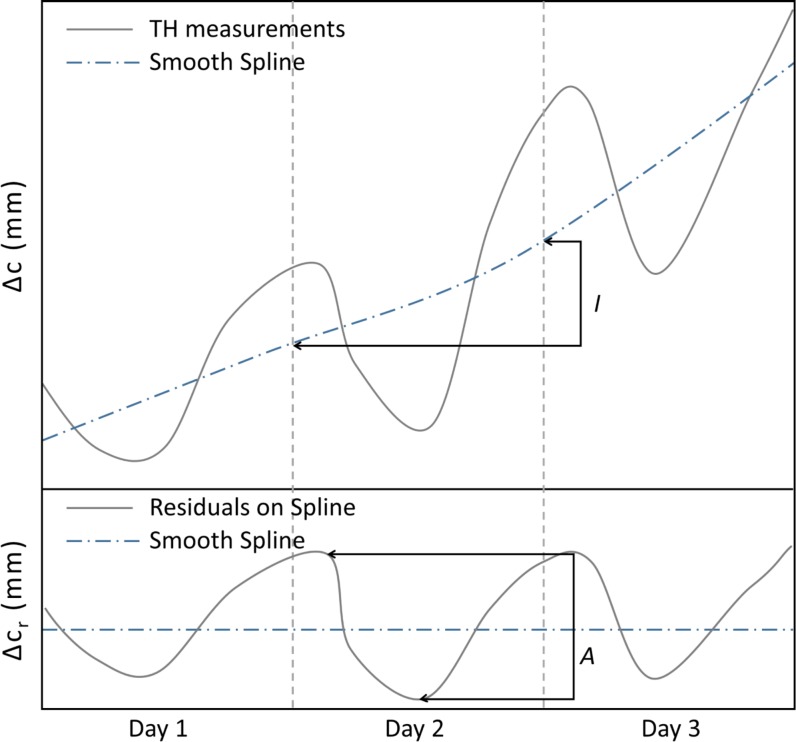
Schematic diagram illustrating metrics of tree circumference change. Shown are the most commonly observed pattern of dynamics in tree circumference change (Δ*c*) and how these are analyzed to calculate daily circumference increment (*I*), residual variation (Δ*c*_*r*_), and daily amplitude (*A*).

From circumference change (*Δc*; mm), daily circumference increment (*I*; mm), residual variation (*Δc*_*r*_), and daily amplitude (*A*; mm) were computed (Fig 2). Δc was calculated relative to the first measurement as Δ*c* = sensor position or window at time *t–*sensor position or window at time 0. To calculate *I* and Δ*c*_*r*_, a smooth spline was fitted to the dendrometer data using the “smooth.spline” function in R with degrees of freedom equal the number of days. The daily circumference increment (*I*) of day *i* was calculated as the difference between the value of the spline at midnight of day *i+1* minus the value of the spline at midnight of day *i*. Residuals between the ADBs measurement and smoothed spline (Δ*c*_*r*_) were calculated to characterize daily patterns. For each day of the study and for each tree, a daily amplitude (*A*) was calculated as the difference between the maximum and minimum values of the residuals (Δ *c*_*r*,*max*_ -Δ*c*_*r*,*min*_; Fig 2).

An ensemble band temperature, *T*_*band*_, was calculated for each site as the median of individual temperature records after removal of outliers. An outlier was a data point located outside 1.5 times the interquartile range above the upper quartile and below the lower quartile of all data points measured at the same time of day. The resulting ensemble estimates of *T*_*band*_ aligned as expected with measurements of *T*_*air*_ at nearby weather stations (see S3 Appendix).

#### Daily patterns

To determine if stems exhibited daily shrinkage, we analyzed how the periodicity of **Δ***c*_*r*_~*Time* and *T*_*band*_~*Time* compared to each other for each tree separately, focusing only on rain-free days. *T*_*band*_ was selected as the focal independent variable for wavelet analysis because it exhibits regular daily cyclicity, is a potential driver of **Δ***c*_*r*_, and was available for all sites. We performed a bivariate wavelet analysis using the “analyze.coherence” function in the “WaveletComp” package in R [43]. We calculated the circular mean phase angle *(θ; rad)* using all phase angles measured for the period equivalent to 1 day and with coherence > 0.7. The coherence is the equivalent of R-squared in a regular linear regression, and the phase angle *θ* is a measure of temporal lag between *T*_*band*_ and **Δ***c*_*r*_. For a tree that is shrinking during the day, *θ* will be between π/2 and 3π/2, indicating that *T*_*band*_ and *Δc*_*r*_ are out of phase (up to 6 hours lag or lead). When *θ* is between π/2 and π, *T*_*band*_ is the leading variable, indicating that temperature may be driving *Δc*_*r*_.

To determine how often each trees exhibited daily shrinkage, we used the same analysis as above but looking at each rain-free day of each tree separately. *θ*_*day*_ was calculated for days where all coherences >0.7. We classified trees as “shrinking” when they exhibited daily shrinking on more than 75% of the days, “swelling” when they were swelling on more than 75% of the days and “no dominant pattern” when they did not have a dominant pattern.

To explore potential drivers of daily shrinkage for the subset of living trees with shrinkage on ≥75% of days, we tested the relationship between *A* (Fig 2) and climatic and several biomicrometeorological variables: daily range of *T*_*band*_, daily average of *VPD*, daily sum of radiation *Rad*, and daily sum of *SF* (SCBI, SERC; each tree was assigned the *SF* record corresponding to its genus) or daily sum of *ET* (BCI, WFDP). For each site separately, we analyzed the correlation between *A* and these variables on rain-free days using a mixed effects model (“lme4” package in R; [44]) with the climatic or biomicrometeorological variable as a fixed effect and tree as a random effect (applied to both intercepts and slopes). The models were tested against a null model that assumed that *A* was constant across the range of climatic or biomicrometeorological variable.

#### Rain responses

To explore the rain-induced changes in daily growth increment *I*, we tested for correlation between *I* and a binomial variable indicating whether *I* occurred on a rainy or a rain-free day. To test if the rain-induced changes were caused by humidification of thick outer bark, we added a categorical variable classifying species as having thin bark (typically <5 mm; e.g., *Fagus grandifolia*, *Taxus brevifolia*, and most species at BCI) or thick bark (e.g., *Liriodendron tulipifera*, *Quercus* spp., *Pseudotsuga menziesii*, *Tabebuia* spp.; S1 Appendix). We tested each site separately using a mixed effects model (“lme4” package in R; [44]) with the binomial variable and/or the bark thickness as a fixed effect(s) and tree as a random effect (applied to both intercepts and slopes of the binomial effect). The models were tested against each other and against a null model that assumed that *I* was not dependent on precipitation or bark thickness. For SERC, we separately tested if trees located in the irrigated plot had a different rain response than the other by including the location (2-level categorical variable for irrigated/not irrigated) in the model as fixed effect.

For all mixed model analysis, we performed a likelihood ratio tests using the R function “anova” and picked the best model based on p-value.

## Results

In total, 83.9% of the total data potentially collected in two months on 127 trees and with 15-minute interval recordings was deemed reliable. This included full or partial records from 108 of 127 trees (S1 Appendix). Full details on ADB performance are presented in S2 Appendix. Henceforth, all results apply only to the records deemed reliable.

### Daily patterns

**Fig 3:**
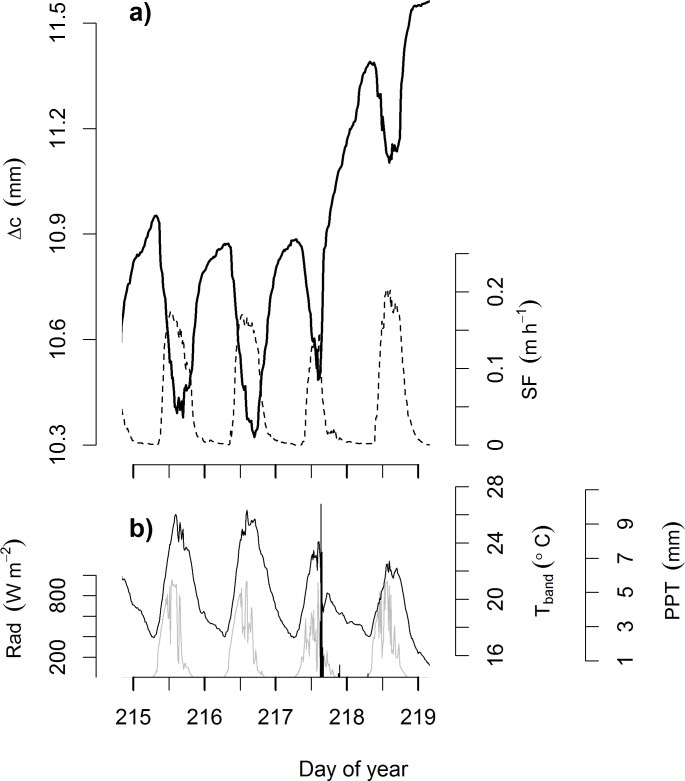
Example record illustrating patterns of tree circumference dynamics that align with physiological expectations. (a) Change in circumference relative to the start of the measurement record (*Δc*; solid line) and sap flow (*SF*; dashed line) measured on a 70 cm DBH *Liriodendron tulipifera* at SCBI; (b) band temperature (*T*_*band*_; black line), solar radiation (*Rad*; grey line), and precipitation (*PPT*; vertical black lines).

As rain events tended to distort daily patterns (Fig 3), all results described in this section refer to non-rain days. Rain-free days occurred on 40% of days at SCBI, 35% of days at SERC, 16% of days at WFDP, and 57% of days at BCI.

**Fig 4:**
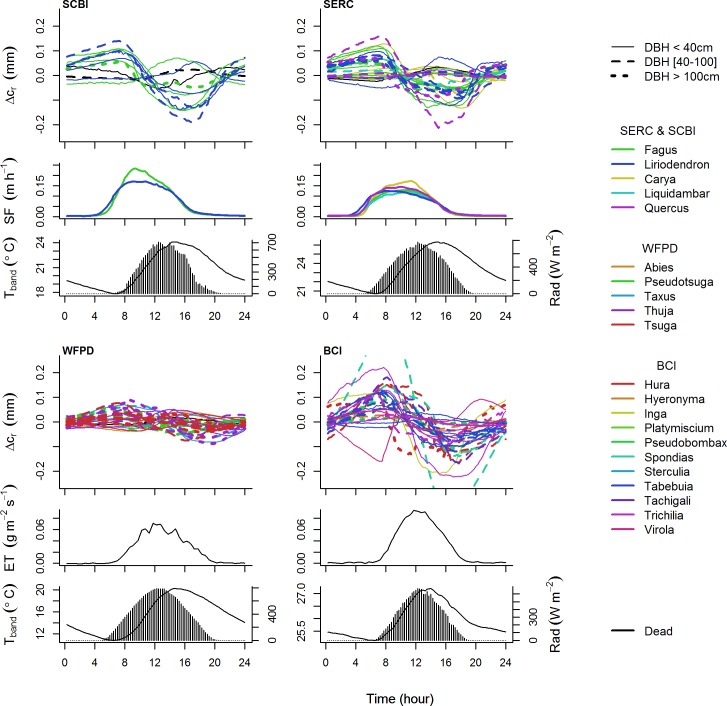
Average daily patterns in stem circumference relative to other variables. Average daily patterns in *Δc*_*r*_, sap flow (*SF*; averaged by genus) or evapotranspiration (ET), temperature (*T*_*band*_), and radiation (*Rad*) on non-rain days during analysis period from the four study sites. For Δ*c*_*r*_, line type indicates size class, and lines are colored by genus as indicated in the legends. *T*_*band*_ is plotted as solid line, *Rad* as bars.

**Fig 5:**
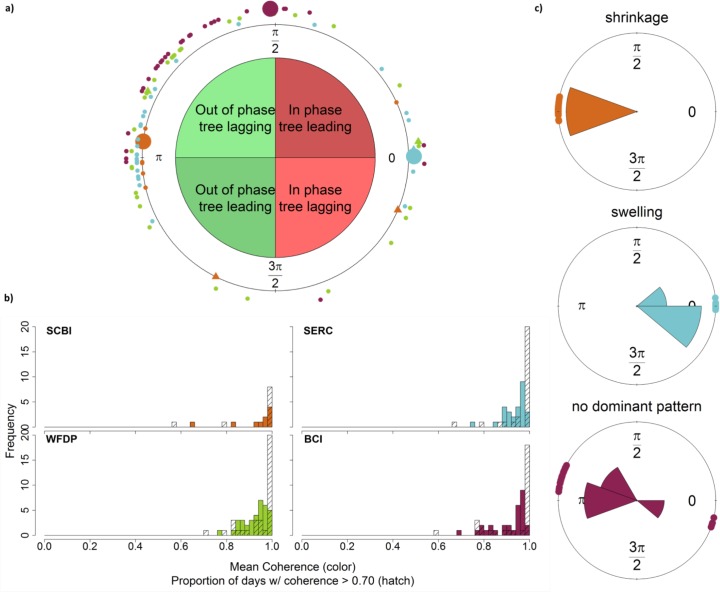
Daily patterns of tree circumference change in relation to temperature. (a) Diagram showing mean phase angle *θ* for all trees (circles: live trees; triangles: dead trees), where π/2 < *θ <* 3π/2 indicates shrinkage and *θ* < π/2 or *θ >* 3π/2 indicates swelling; (b) Histograms of mean coherence (colored bars) and proportion of days with coherence greater than 70% (hatched bars); (c) examples of daily (as opposed to mean) phase angles (*θ*_*day*_) for individual trees, corresponding to larger symbols in (a). Examples include a tree with shrinkage on ≥75% of days (*Liriodendron tulipifera*, DBH = 49.3 cm, SCBI), one with swelling ≥75% of days (*Liriodendron tulipifera*, DBH = 69.3, SERC), and one with no consistent pattern (*Hura crepitans*, DBH = 67.1 cm, BCI). For all 3 panels site-colors correspondences are given in (b).

The majority of living trees exhibited diurnal shrinkage, both on average and on the majority of days; however, diurnal expansion and lack of a consistent pattern were also common (Figs 4 and 5). The wavelet analysis showed that the majority of living trees (63.7%) had a mean phase angle indicating daily shrinkage, whereas others exhibited daily expansion (Fig 5A). These mean phase angles were sometimes representative of a consistent pattern on individual days, but sometimes obscured opposite patterns, where a tree would shrink on some days and swell on others (Fig 5C). For most trees, coherence (similar to R^2^) was >0.70 on >80% of the days, indicating that pronounced daily patterns were common, albeit sometimes switching direction (Fig 5B).

The analysis focused on phase angle by individual days (as opposed to mean phase angle) revealed that individual trees commonly exhibited a mix of shrinking or swelling (Fig 5C, Table 3). Of the live trees at all sites combined, 64% exhibited daily shrinkage on ≥75% of days, whereas 13% exhibited significant daily expansion on ≥75% of days and 23% had no dominant pattern (Table 3). Patterns varied by site, with 70% of live trees at SCBI, 63% of trees at SERC, 54% of trees at WFDP, and 73% of trees at BCI exhibiting daily shrinkage on more than 75% of the days. Of the dead trees, three exhibited daily expansion, one exhibited daily shrinkage (at WFDP), and one exhibited no dominant pattern (at SCBI).

**Table 3:** Numbers and mean daily amplitude (*A*) of ADBs exhibiting daily shrinkage, expansion, or no dominant pattern.

		all		Shrinkage ≥75% of days		Expansion ≥75% of days		No dominant pattern	
Category	Site	n	*A* ± SD	n	*A* ± SD	n	*A* ± SD	n	*A* ± SD
Live trees	SCBI	10	0.23 ± 0.07	7	0.25 ± 0.07	1	0.15	2	0.21 ± 0.07
	SERC	27	0.15 ± 0.10	17	0.20 ± 0.08	5	0.07 ± 0.05	5	0.05 ± 0.03
	WFDP	35	0.01 ± 0.04	19	0.11 ± 0.05	6	0.08 ± 0.01	10	0.09 ± 0.03
	BCI	30	0.26 ± 0.16	22	0.29 ± 0.17	1	0.03	7	0.25 ± 0.14
	all	102	0.15 ± 0.15	65	0.21 ± 0.13	13	0.08 ± 0.04	24	0.14 ± 0.11
Dead trees	all	5	0.08 ± 0.06	1	0.07	3	0.07 ± 0.01	1	0.16
Glass vases^a^	-	2	0.00 ± 0.00	0	-	0	-	2	0.00 ± 0.00

Bands are classified based on wavelet analysis of the relationship of Δ*c*_*r*_ to *T*_*band*_.

^a^See S4 Appendix

For all live trees combined, the amplitude of daily circumference variation, *A*, averaged 0.15 mm (SD = 0.15 mm) and ranged from 0.01–1.92 mm (Table 3). There was a tendency for trees exhibiting shrinkage on ≥75% of days to have larger *A* (mean = 0.20, SD = 0.13) than others (mean = 0.12, SD = 0.09; t-test, t(90) = 4, p < 0.001). The amplitude of daily signals varied by site (ANOVA, F(3) = 18.93, p < 0.001). A post-hoc pairwise t-test showed that the WFDP (mean = 0.09, SD = 0.06) was the site with the smallest daily amplitudes and significantly differed from the three other sites. BCI had the largest daily amplitudes (mean = 0.26, SD = 0.25), differing significantly from SERC (mean = 0.15, SD = 0.12) and WFDP, but not SCBI (mean = 0.23, SD = 0.11; Table 3). We did not find any significant relationship between *A* and DBH (mixed model with site as random effect, χ^2^(1) = 1.74, p = 0.19, indicating either that larger trees shrink less in proportion to their size or that friction of the band against the bark prevents full tightening of the band around the tree on a daily basis.

For the subsets of trees with shrinkage on ≥75% of days, *A* was significantly correlated with one or more climatic and biomicrometeorological variables at all four sites (Table 4). When there was a significant relationship, random and fixed effects always explained more than 37% of the variance (Table 4) and all trees had same sign of slope (not shown). Specifically, *A* was positively correlated with the daily range of *T*_*band*_ (all p ≤ 0.03) at all sites. There was a positive correlation between *A* and daily average VPD at SERC and WFDP (both p ≤0.001), but not at SCBI or BCI (both p ≥ 0.193). *A* was positively correlated with the daily radiation sum at SERC, WFDP and BCI (all p ≤ 0.001), but not at SCBI (p = 0.11). There was a significant correlation between *A* and daily *SF* at SERC (p = 0.03) but not at SCBI (p = 0.364). *A* was significantly correlated with daily *ET* at both WFDP and BCI (both p ≤ 0.001).

**Table 4:** Mixed model results for correlations between *A* (of trees with shrinkage on ≥75% of days) and scaled daily values of climatic and biomicrometeorological variables.

Site	n obs	n trees	AIC	AICnull	ΔAIC	likelihood ratio test		slope		intercept		% variance explained	
						Χ^2^ (df)	p	est ± SD	*t*	est ± SD	*t*	Fixed effect only	Fixed & random effects
**Daily range *T***_***band***_													
SCBI	254	7	-516	-511	5	7.02(1)	0.008	0.02 ± 0.01	3.21	0.25 ± 0.03	9.64	3.25	41.94
SERC	629	17	-1187	-1175	12	13.25(1)	<0.001	0.02 ± 0.00	4.50	0.21 ± 0.02	10.42	2.77	46.57
WFDP	976	19	-3306	-3292	16	15.61(1)	<0.001	0.02 ± 0.00	4.92	0.11 ± 0.01	10.64	7.38	57.81
BCI	555	22	-106	-102	4	5.12(1)	0.024	0.02 ± 0.01	2.28	0.29 ± 0.03	8.28	0.59	37.13
**Daily avg *VPD***													
SCBI	254	7	-504	-504	0	0.17(1)	0.678	-	-	0.24 ± 0.02	9.84	-	-
SERC	629	17	-1243	-1229	14	15.22(1)	<0.001	0.03 ± 0.01	5.12	0.21 ± 0.02	16.99	6	52.13
WFDP	976	19	-3543	-3529	14	15.22(1)	<0.001	0.02 ± 0.00	4.83	0.11 ± 0.01	19.01	12.47	67.58
BCI	555	22	-287	-187	0	2.07(1)	0.150	-	-	0.26 ± 0.03	8.48	-	-
**Daily sum *Rad***														
SCBI	254	7	-502	-502	0	1.99(1)	0.158	-	-	0.25 ± 0.03	9.62	-	-
SERC	629	17	-1219	-1207	12	13.61(1)	<0.001	0.02 ± 0.01	4.53	0.21 ± 0.02	16.98	4.11	49.6
WFDP	976	19	3431	-3418	13	15.53(10)	<0.001	0.02 ± 0.00	4.90	0.11 ± 0.01	19.01	10.29	63.25
BCI	555	22	-150	-142	8	10.17(1)	0.001	0.04 ± 0.01	3.54	0.29 ± 0.03	22.15	2.79	42.16
**Daily sum *SF***													
SCBI	226	7	-438	-438	0	0.82 (1)	0.364	-	-	0.24 ± 0.02	11.05	-	-
SERC	495	17	-978	-972	6	8.55(1)	0.003	0.02 ± 0.01	3.34	0.21 ± 0.02	17.01	3.38	51.15
**Daily sum *ET***													
WFDP	976	19	-3344	-3329	15	16.39(1)	<0.001	0.02 ± 0.00	5.10	0.11 ± 0.01	19.01	8.57	59.49
BCI	555	22	-130	-117	13	14.06(1)	<0.001	0.04 ± 0.01	4.16	0.29 ± 0.03	22.16	2.48	39.78

### Rain responses

**Fig 6:**
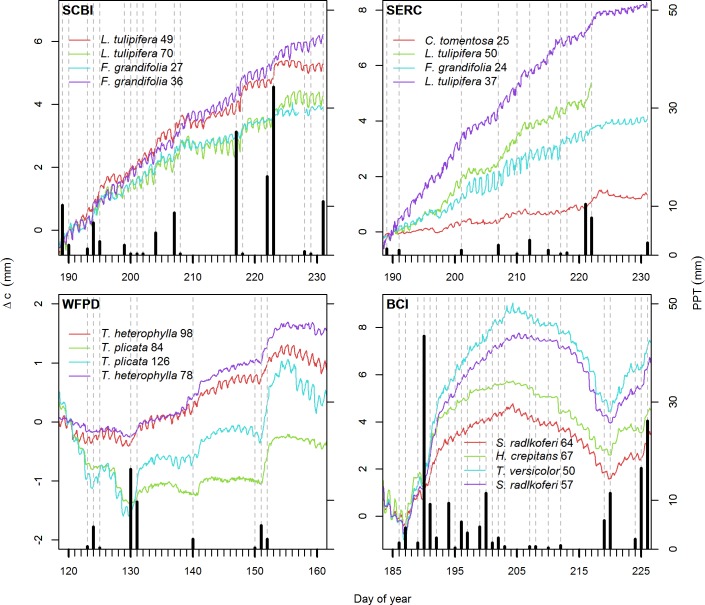
Example responses of trees at each of the four study sites to rain events. Vertical dashed grey lines are rainy days and vertical solid black lines are precipitation amounts. Each colored line represent one tree, with species and DBH (cm) indicated in legends. Trees were selected for relatively strong rain responses and good data records over a 40-day time period selected to focus on rain events.

Trees commonly responded to rain events with notably higher *I* (Figs 3 and 6; Table 5). Having rain as a categorical fixed effect always improved the mixed model (all p < 0.001), and the estimated differences between *I* on rainy days versus *I* on rain free days ranged from +0.04 mm to +0.16 mm. At two sites, (WFDP and BCI), trees tended to have negative *I* on rain-free days and positive *I* on rainy days (Table 5). Bark thickness was never a significant fixed effect in the mixed linear models (all p > 0.43). For SERC, including the categorical variable indicating whether the trees were in the irrigated plot or not did not improve the model (χ^2^(1) < 0.00, p = 0.99), indicating that the watering experiment had no detectable effect on the rain response. Dead trees, four out of five of which had bark, did not respond significantly to rain events (p = 0.23).

**Table 5:** Mixed model results for rain effect on *I*.

		n obs	n trees	AIC	AICnull	ΔAIC	p	Estimate of the difference between *I* on rainy vs rain free day		Estimate of *I* on rain free day	
Category	Site							est ± SD	*t*	est ± SD	*t*
Live	SCBI	538	10	-901	-881	20	<0.001	0.12 ± 0.01	8.55	0.06 ± 0.02	3.9
	SERC	1558	27	-3403	-3390	13	<0.001	0.04 ± 0.01	4.31	0.02 ± 0.01	2.6
	WFDP	2106	35	-5356	-5341	15	<0.001	0.04 ± 0.01	4.64	-0.01 ± 0.01	-2
	BCI	1575	29	-472	-452	20	<0.001	0.16 ± 0.03	5.61	-0.04 ± 0.01	-3.08
Dead	All	285	5	-540	-540	0	0.228	-	-	-0.01 ± 0.02	-0.38

## Discussion

Trees undergo daily cycles of circumference fluctuation that are most often, but not always, consistent with expectations based on known physiological mechanisms [20,45] (Figs 3–5, Table 3), and they commonly exhibit circumference changes in response to rain events (Figs 3 and 6; Table 5). As detailed below, our results indicate that on the daily time scale, over-bark measurements of tree circumference dynamics have limited potential for estimating xylem water storage or transpiration, and daily circumference variations are not large enough to be of concern for most studies estimating tree growth. On time scales of days to weeks, stem shrinkage and swelling occurs in response to changes in water availability, and these can be large enough to potentially bias estimates of tree growth and forest productivity (Fig 6).

### Daily circumference variation

The daily patterns of circumference variation recorded by ADBs were frequently—but not consistently—shaped by changes in stem water content. The most commonly observed pattern of daily circumference variation in live trees was shrinkage (Figs 4 and 5; Table 3), which is consistent with numerous observations that changes in stem hydraulic status result in daytime shrinkage of xylem, phloem, and inner bark [13,16,20,23]. Daily shrinkage typically began at or shortly after sunrise, aligning with the timing of the onset of transpiration (*SF* or *ET*; Figs 3 and 4). Trees commonly reached minimum circumference in the late afternoon before expanding overnight to new pre-dawn maxima—a pattern that aligns with physiological expectations [20,21]. Furthermore, for trees with shrinkage patterns on ≥75% of the days, the fact that shrinkage tended to be greater (larger *A*)—often significantly so—on days with higher VPD, radiation, sap flow or evapotranspiration (Table 4) is consistent with previous observations of greater shrinkage on days when trees are more actively transpiring [45,46]. Thus, it appears that a hydrological mechanism was often a driver of the daily circumference variation observed here; however, as discussed below, our results also point to the influence of other mechanisms.

Besides stem hydraulics, there are two potential alternative mechanisms for daily shrinkage patterns that have minimal effects on point dendrometers but may strongly influence ADBs: thermal expansion of the bands and humidity-driven swelling of outer bark. Because stainless steel expands and contracts with changes in temperature, daily temperature cycles could result in an artificial shrinkage pattern as warmer midday temperatures create slack in the band. However, the physics of the system is complex and may be mediated by friction or counterbalanced by thermal expansion of the trees [47,48]. We did record consistent diurnal shrinkage on some trees for which a hydraulic mechanism is unreasonable, including one dead tree and some trees with very thick bark (e.g., *Pseudotsuga menziesii*, some individuals of which had >10 cm thick bark). However, the relatively small amplitude of this shrinkage (Table 3; Fig 4), the lack of consistent daily shrinkage patterns for most dead trees (Table 3), and our thermal experiment (S4 Appendix) indicate that a thermal mechanism is insufficient to explain the frequency and magnitude of observed diurnal shrinkage. Moreover, the fact that trees on BCI had the largest average *A* despite very small daily temperature variations (<2°C; Fig 3, Table 3) is a good indication that temperature is unlikely to be the driver of observed diurnal shrinkages. It is also possible that diurnal shrinkage patterns could be influenced by humidity-driven increases in the hydration of outer bark at night. While this may explain diurnal shrinkage of trees with very thick bark (e.g., *Pseudotsuga menziesii*, Fig 4), pronounced shrinkage on many thin-barked species (e.g., *Fagus grandifolia*; many species at BCI; Fig 4) indicates that this mechanism is not sufficient to explain observed patterns. Thus, while thermal expansion of ADBs and humidity-driven swelling of bark may have played a role in the observed patterns, neither is sufficient to explain observed shrinkage patterns.

While daily shrinkage was the most frequently observed pattern, expansion was also common, albeit typically with lower *A* than shrinkage (Figs 4 and 5; Table 3). The most probable explanation for this is thermal expansion of the trees, which has been observed [47,48]. Midday stem expansion is not consistent with any known hydraulic mechanism [20], although it should tend to be greatest when not counteracted by strong diurnal patterns in stem water content. Thus, our results indicate that ADB signals are influenced by a complex interplay of hydraulic and thermal mechanisms. The use of ADBs to estimate transpiration rates would therefore require extensive further study and calibration.

Our results indicate that diurnal variations in stem circumference are unlikely to bias estimates of tree growth and forest productivity. Daily circumference variation was typically <0.5 mm (0.16 mm diameter), averaging 0.15 ±0.15 mm (Table 3) and exceeding a daily average of >0.5 mm on only one tree (a 136 cm DBH *Pseudobombax septenatum* on BCI; Fig 3). Thus, daily circumference variation introduces only limited potential for error in estimation of tree growth rates, and should be of concern only to studies using manual dendrometer bands to characterize growth rates over very short time frames (i.e., daily), on slowly growing trees, or on trees with large *A*.

### Rain responses

Trees commonly responded to rain events with increases in the daily circumference increment, *I* (Figs 3 and 6; Table 5). This response tended to be particularly pronounced following periods of little rain (Fig 6). In particular, on BCI, where a major El Niño event resulted in unusually dry conditions, some trees expanded several millimeters circumference over the course of a few days following a large rain event on July 11 (Day of Year 189; Fig 6). Rain responses were likely a combination of enlargement of living cells driven by increased water pressure [49] and hydration of the outer bark [32]. While the former mechanism was almost certainly important at BCI, where most instrumented trees had relatively thin bark, the latter was undoubtedly dominant on very thick-barked species at WFDP (e.g., *Pseudotsuga menziesii*). The fact that we did not detect differences in rain responses between thin- and thick- barked species and the lack of rain responses in dead trees indicates that expansion of living tissues are at least partially responsible for rain responses. Meanwhile, the fact that we detected no differences in rain responses between trees in watered and unwatered plots at SERC suggests that the influence of soil moisture on water storage in living tissues was small relative to bark hydration. To the extent that rain responses represent differences in stem, as opposed to outer bark, hydration, the signals of ADBs can provide a metric of tree water status. Studies using point dendrometers have revealed that such diameter dynamics provide a useful metric of tree water stress [12,15]. Given the relatively short time scales over which tree stems vary in response to water availability, capturing these responses is among the most useful capabilities of ADBs.

For the trees studied here, rain-driven stem expansion was of a magnitude that could introduce minimal to modest error into estimates of tree growth (Fig 4). Stem expansion over the course of 10 days following a large rain event on July 11 at BCI (Fig 6) ranged up to 5.04 mm for a 50.2 cm DBH *Tachigali versicolor*, and some of this tree’s expansion following rain was later reversed during a period of little rain (blue line in BCI plot of Fig 4). Rain responses such as this are substantial relative to annual stem growth. Based on census data from the CTFS-ForestGEO plot on BCI (downloaded Feb. 29, 2016 from http://ctfs.si.edu/ctfsrep; [38,39,50]), the mean annual diameter growth of 36.5–66.5 cm DBH individuals of this species was 17.38 mm yr^-1^; thus, an increase of 5.04 mm in circumference can represent >9% of the annual growth of the tree. It is therefore important that researchers working with stem diameter data be aware of potential introduction of error by rain, particularly for sites with variable water availability where rain responses can be large [51]. Bias may be avoided by standardizing the timing of manual measurements with respect to rain events or by making manual or automated measurements at regular, relatively high-frequency intervals and including rain events in interpretation of the data (e.g., [7]).

### Automated dendrometer bands as a research tool

ADBs hold mixed potential as a research tool for forest ecophysiology. They are attractive in that they are non-invasive, easy to install, and generally do not require an external power source or data logger. Cost, reliability, and ease of maintenance are important considerations that vary by model and should be expected to evolve with the technology. Overall, ADBs are one of the more accessible technologies for automated measurements related to tree ecophysiology. However, a major drawback is that the signals of ADBs do not consistently translate into meaningful metrics of tree physiology. Over-the-bark installation can modify or obscure the signals from living tissues, thermal expansion of the band or tree may influence daily circumference dynamics, and friction between the bark and the band has uncertain effects. Experiments focused on disentangling how environmental conditions interact with the physics of the band and the tree would be useful for interpreting the patterns detected by ADBs. To the extent that the influence of the outer bark and frictional forces can be empirically corrected or modeled, ADB data could be valuable for constraining mechanistic tree hydraulic models [52,53], providing direct metrics of tree hydraulic status [16,25], or correcting growth measurements for the influence of stem hydration.

## Conclusions

Fine-scale stem circumference dynamics recorded by ADBs commonly reflect tree water status and hold some potential for characterizing forest ecophysiology. While stem shrinkage patterns do not translate to reliable estimates of transpiration on the scale of forest stands, the short-term dynamics of tree circumference shrinking and swelling in response to dry spells and rain events, respectively, may serve as a metric of tree hydraulic stress and be useful for disentangling seasonal dynamics of stem growth in environments with variable water availability. In terms of improving estimates of tree growth and forest productivity, high frequency measurements are not necessary to standardize for circumference variations on the daily time scale, but could prove useful for distinguishing stem growth from reversible hydraulic expansion in environments with strong variation in water availability.

## Supporting Information

S1 Appendix List of all trees equipped with ADBs. (DOCX)

S2 Appendix TreeHugger failures. (DOCX)

S3 Appendix Relationship between *T*_*band*_ and *T*_*air*_. (DOCX)

S4 Appendix Thermal expansion experiment. (DOCX)
