# Supplementary material for: Tree Circumference Dynamics in Four Forests Characterized Using Automated Dendrometer Bands
Source: PLoS One. 2016 Dec 28;11(12):e0169020. doi: 10.1371/journal.pone.0169020 (PMC5193451; doi:10.1371/journal.pone.0169020)
Supplement: S1 Appendix — (DOCX) [file pone.0169020.s001.docx]

**Tree circumference dynamics over short time scales using automated dendrometer bands in four forests**

Valentine Herrmann, Sean M. McMahon, Matteo Detto, James A. Lutz, Stuart J. Davies, Chia-Hao Chang-Yang, Kristina J. Anderson-Teixeira

# S1 Table. List of all trees equipped with ADBs

| **Site** | **Band number** | **Species** | **Band diameter (cm)** | **Bark Thickness** | **Watered** | **Date equipped** | **Good data (%)** |
| --- | --- | --- | --- | --- | --- | --- | --- |
| BCI | TH14230 | Tabebuia guayacan | 23.3 | Thick | NO | 6/25/2015 | 76 |
| BCI | TH14231 | Pseudobombax septenatum | 136.1 | Thin | NO | 6/24/2015 | 73 |
| BCI | TH14234 | Spondias radlkoferi | 63.8 | Thin | NO | 6/25/2015 | 100 |
| BCI | TH14236 | Hura crepitans | 137.5 | Thin | NO | 6/24/2015 | 75 |
| BCI | TH14239 | Tabebuia rosea | 39.3 | Thick | NO | 6/24/2015 | 100 |
| BCI | TH14244 | Trichilia tuberculata | 21 | Thin | NO | 6/23/2015 | 100 |
| BCI | TH14245 | Tabebuia rosea | 22.3 | Thick | NO | 6/24/2015 | 81 |
| BCI | TH14248 | Tachigali versicolor | 50.2 | Thin | NO | 6/24/2015 | 86 |
| BCI | TH14252 | Hyeronyma alchorneoide | 69.5 | Thin | NO | 6/25/2015 | 100 |
| BCI | TH14253 | Trichilia tuberculata | 34.9 | Thin | NO | 6/24/2015 | 89 |
| BCI | TH14255 | Platymiscium pinnatum | 50.6 | Thick | NO | 6/24/2015 | 0 |
| BCI | TH14256 | Tabebuia guayacan | 54.4 | Thick | NO | 6/23/2015 | 97 |
| BCI | TH14261 | Hura crepitans | 67.1 | Thin | NO | 6/24/2015 | 100 |
| BCI | TH14264 | Inga pezizifera | 33.5 | Thin | NO | 6/23/2015 | 88 |
| BCI | TH14266 | Tabebuia rosea | 33.8 | Thick | NO | 6/25/2015 | 87 |
| BCI | TH14269 | Virola sebifera | 34.5 | Thin | NO | 6/26/2015 | 70 |
| BCI | TH14270 | Tabebuia guayacan | 80.3 | Thick | NO | 6/24/2015 | 100 |
| BCI | TH14271 | Hura crepitans | 91.1 | Thin | NO | 6/24/2015 | 0 |
| BCI | TH14274 | Spondias radlkoferi | 74.1 | Thin | NO | 6/25/2015 | 100 |
| BCI | TH14275 | Virola sebifera | 22 | Thin | NO | 6/26/2015 | 100 |
| BCI | TH14277 | Trichilia tuberculata | 42.25 | Thin | NO | 6/24/2015 | 86 |
| BCI | TH14279 | Trichilia tuberculata | 27.4 | Thin | NO | 6/23/2015 | 95 |
| BCI | TH14281 | Tabebuia rosea | 51.2 | Thick | NO | 6/25/2015 | 98 |
| BCI | TH14283 | Tabebuia guayacan | 59 | Thick | NO | 6/24/2015 | 98 |
| BCI | TH14284 | Spondias radlkoferi | 57.1 | Thin | NO | 6/25/2015 | 78 |
| BCI | TH14285 | Hura crepitans | 98 | Thin | NO | 6/25/2015 | 62 |
| BCI | TH14288 | Pseudobombax septenatum | 102.4 | Thin | NO | 6/25/2015 | 100 |
| BCI | TH14289 | Tabebuia rosea | 59 | Thick | NO | 6/22/2015 | 97 |
| BCI | TH14291 | Spondias radlkoferi | 32.2 | NA | NO | 6/25/2015 | 95 |
| BCI | TH14294 | Trichilia tuberculata | 30 | Thin | NO | 6/23/2015 | 55 |
| BCI | TH14299 | Virola sebifera | 29.9 | Thin | NO | 6/24/2015 | 100 |
| BCI | TH14303 | Tabebuia rosea | 34 | Thick | NO | 6/24/2015 | 100 |
| BCI | TH14304 | Virola surinamensis | 64.4 | Thin | NO | 6/23/2015 | 0 |
| BCI | TH14305 | Tabebuia guayacan | 21.6 | Thick | NO | 6/24/2015 | 76 |
| SCBI | TH30233 | Fagus grandifolia | 105.89 | Thin | NO | 5/5/2014 | 90 |
| SCBI | TH33392 | Liriodendron tulipifera | 49.34 | Thick | NO | 5/5/2014 | 91 |
| SCBI | TH33393 | Liriodendron tulipifera | 14.87 | Thick | NO | 5/5/2014 | 100 |
| SCBI | TH40563 | Fagus grandifolia | 36.4 | Thin | NO | 5/5/2014 | 100 |
| SCBI | TH40568 | Fagus grandifolia | 16.94 | Thin | NO | 5/5/2014 | 98 |
| SCBI | TH40569 | Liriodendron tulipifera | 28.9 | Thick | NO | 5/5/2014 | 62 |
| SCBI | TH40579 | Dead | 62.6 | Thick | NO | 5/5/2014 | 100 |
| SCBI | TH50650 | Liriodendron tulipifera | 18 | Thick | NO | 5/5/2014 | 100 |
| SCBI | TH50654 | Liriodendron tulipifera | 69.98 | Thick | NO | 5/5/2014 | 100 |
| SCBI | TH60453 | Dead | 19.8 | Thin | NO | 5/5/2014 | 73 |
| SCBI | TH60458 | Fagus grandifolia | 26.7 | Thin | NO | 5/5/2014 | 98 |
| SCBI | TH60462 | Fagus grandifolia | 20.73 | Thin | NO | 5/5/2014 | 56 |
| SERC | TH14000 | Carya glabra | 19.4 | Thick | NO | 6/15/2015 | 0 |
| SERC | TH14225 | Quercus alba | 32 | Thick | NO | 6/15/2015 | 100 |
| SERC | TH14226 | Fagus grandifolia | 44.75 | Thin | YES | 6/15/2015 | 0 |
| SERC | TH14227 | Carya tomentosa | 24.9 | Thick | YES | 6/15/2015 | 100 |
| SERC | TH14228 | Carya tomentosa | 57.4 | Thick | NO | 6/15/2015 | 97 |
| SERC | TH14229 | Carya tomentosa | 20.4 | Thick | NO | 6/15/2015 | 98 |
| SERC | TH14232 | Quercus falcata | 73.4 | Thick | NO | 6/15/2015 | 0 |
| SERC | TH14233 | Liriodendron tulipifera | 37.1 | Thick | NO | 6/15/2015 | 100 |
| SERC | TH14235 | Quercus falcata | 111.6 | Thick | YES | 6/15/2015 | 78 |
| SERC | TH14237 | Carya tomentosa | 26.4 | Thick | YES | 6/15/2015 | 0 |
| SERC | TH14238 | Liriodendron tulipifera | 69.3 | Thick | NO | 6/15/2015 | 100 |
| SERC | TH14240 | Fagus grandifolia | 23.7 | Thin | NO | 6/15/2015 | 100 |
| SERC | TH14241 | Liquidambar styraciflua | 60 | Thick | YES | 6/15/2015 | 78 |
| SERC | TH14242 | Quercus falcata | 44.1 | Thick | NO | 6/15/2015 | 87 |
| SERC | TH14243 | Fagus grandifolia | 27.8 | Thin | YES | 6/15/2015 | 100 |
| SERC | TH14246 | Fagus grandifolia | 34.2 | Thin | YES | 6/15/2015 | 84 |
| SERC | TH14247 | Fagus grandifolia | 25.8 | Thin | YES | 6/15/2015 | 100 |
| SERC | TH14251 | Liquidambar styraciflua | 57.7 | Thick | NO | 6/15/2015 | 0 |
| SERC | TH14259 | Fagus grandifolia | 35.4 | Thin | NO | 6/15/2015 | 100 |
| SERC | TH14263 | Dead | 36.4 | Thick | YES | 6/15/2015 | 100 |
| SERC | TH14265 | Acer rubra | 19.1 | Thin | NO | 6/15/2015 | 0 |
| SERC | TH14267 | Carya tomentosa | 28.9 | Thick | NO | 6/15/2015 | 100 |
| SERC | TH14272 | Fagus grandifolia | 28.7 | Thin | NO | 6/15/2015 | 0 |
| SERC | TH14273 | Fagus grandifolia | 47 | Thin | NO | 6/15/2015 | 100 |
| SERC | TH14278 | Liriodendron tulipifera | 50.1 | Thick | NO | 6/15/2015 | 85 |
| SERC | TH14282 | Fagus grandifolia | 34.8 | Thin | NO | 6/15/2015 | 100 |
| SERC | TH14286 | Liriodendron tulipifera | 41 | Thick | NO | 6/15/2015 | 100 |
| SERC | TH14287 | Liriodendron tulipifera | 102 | Thick | YES | 6/15/2015 | 100 |
| SERC | TH14292 | Fagus grandifolia | 16.15 | Thin | NO | 6/15/2015 | 100 |
| SERC | TH14293 | Liriodendron tulipifera | 65 | Thick | YES | 6/15/2015 | 100 |
| SERC | TH14295 | Quercus sp. | 68.3 | Thick | NO | 6/15/2015 | 80 |
| SERC | TH14297 | Fagus grandifolia | 48.9 | Thin | NO | 6/15/2015 | 100 |
| SERC | TH14300 | Liriodendron tulipifera | 17.1 | Thick | NO | 6/15/2015 | 98 |
| SERC | TH14301 | Liquidambar styraciflua | 33.6 | Thick | NO | 6/15/2015 | 80 |
| SERC | TH14306 | Carya tomentosa | 37.5 | Thick | NO | 6/15/2015 | 100 |
| WFPD | TH14031 | Pseudotsuga menziessi | 115 | Thick | NO | 4/21/2015 | 100 |
| WFPD | TH14032 | Pseudotsuga menziessi | 59.5 | Thick | NO | 4/20/2015 | 100 |
| WFPD | TH14033 | Dead | 57.2 | Thick | NO | 4/20/2015 | 100 |
| WFPD | TH14034 | Pseudotsuga menziessi | 122.5 | Thick | NO | 4/21/2015 | 100 |
| WFPD | TH14035 | Pseudotsuga menziessi | 168.2 | Thick | NO | 4/21/2015 | 100 |
| WFPD | TH14036 | Tsuga heterophylla | 97.7 | Thick | NO | 4/21/2015 | 100 |
| WFPD | TH14037 | Pseudotsuga menziessi | 73.7 | Thick | NO | 4/21/2015 | 100 |
| WFPD | TH14038 | Thuja plicata | 30.2 | Thick | NO | 4/20/2015 | 100 |
| WFPD | TH14039 | Pseudotsuga menziessi | 107 | Thick | NO | 4/21/2015 | 100 |
| WFPD | TH14040 | Thuja plicata | 105.6 | Thick | NO | 4/21/2015 | 100 |
| WFPD | TH14041 | Dead | 20.1 | Thick | NO | 4/20/2015 | 100 |
| WFPD | TH14042 | Abies amabilis | 51.7 | Thick | NO | 4/20/2015 | 100 |
| WFPD | TH14043 | Thuja plicata | 180.7 | Thick | NO | 4/21/2015 | 100 |
| WFPD | TH14044 | Abies amabilis | 25.7 | Thick | NO | 4/20/2015 | 100 |
| WFPD | TH14045 | Pseudotsuga menziessi | 87.8 | Thick | NO | 4/20/2015 | 100 |
| WFPD | TH14046 | Tsuga heterophylla | 128.8 | Thick | NO | 4/21/2015 | 79 |
| WFPD | TH14047 | Abies amabilis | 14.4 | Thick | NO | 4/20/2015 | 100 |
| WFPD | TH14048 | Taxus brevifolia | 41.8 | Thin | NO | 4/21/2015 | 100 |
| WFPD | TH14049 | Thuja plicata | 83.7 | Thick | NO | 4/21/2015 | 100 |
| WFPD | TH14050 | Thuja plicata | 49.7 | Thick | NO | 4/20/2015 | 72 |
| WFPD | TH14051 | Thuja plicata | 38.7 | Thick | NO | 4/20/2015 | 100 |
| WFPD | TH14052 | Thuja plicata | 19.6 | Thick | NO | 4/20/2015 | 100 |
| WFPD | TH14053 | Thuja plicata | 68 | Thick | NO | 4/21/2015 | 100 |
| WFPD | TH14054 | Abies amabilis | 46.4 | Thick | NO | 4/21/2015 | 100 |
| WFPD | TH14055 | Tsuga heterophylla | 27.8 | Thick | NO | 4/20/2015 | 100 |
| WFPD | TH14056 | Pseudotsuga menziessi | 143.4 | Thick | NO | 4/20/2015 | 100 |
| WFPD | TH14057 | Dead | 128.1 | Thick | NO | 4/21/2015 | 0 |
| WFPD | TH14058 | Taxus brevifolia | 33.9 | Thin | NO | 4/20/2015 | 100 |
| WFPD | TH14059 | Thuja plicata | 126.1 | Thick | NO | 4/21/2015 | 100 |
| WFPD | TH14060 | Tsuga heterophylla | 16 | Thick | NO | 4/20/2015 | 100 |
| WFPD | TH14121 | Tsuga heterophylla | 39 | Thick | NO | 4/20/2015 | 100 |
| WFPD | TH14122 | Taxus brevifolia | 23.1 | Thin | NO | 4/20/2015 | 100 |
| WFPD | TH14123 | Tsuga heterophylla | 77 | Thick | NO | 4/21/2015 | 100 |
| WFPD | TH14124 | Pseudotsuga menziessi | 135.7 | Thick | NO | 4/21/2015 | 0 |
| WFPD | TH14125 | Tsuga heterophylla | 101.1 | Thick | NO | 4/21/2015 | 100 |
| WFPD | TH14126 | Taxus brevifolia | 14.9 | Thin | NO | 4/20/2015 | 100 |
| WFPD | TH14127 | Taxus brevifolia | 46.3 | Thin | NO | 4/20/2015 | 0 |
| WFPD | TH14128 | Abies amabilis | 38.7 | Thick | NO | 4/20/2015 | 100 |
| WFPD | TH14129 | Tsuga heterophylla | 77.7 | Thick | NO | 4/21/2015 | 100 |
| WFPD | TH14130 | Tsuga heterophylla | 55.6 | Thick | NO | 4/20/2015 | 100 |
