# Supplementary material for: Tree Circumference Dynamics in Four Forests Characterized Using Automated Dendrometer Bands
Source: PLoS One. 2016 Dec 28;11(12):e0169020. doi: 10.1371/journal.pone.0169020 (PMC5193451; doi:10.1371/journal.pone.0169020)
Supplement: S2 Appendix — (DOCX) [file pone.0169020.s002.docx]

**Tree circumference dynamics over short time scales using automated dendrometer bands in four forests**

Valentine Herrmann, Sean M. McMahon, Matteo Detto, James A. Lutz, Stuart J. Davies, Chia-Hao Chang-Yang, Kristina J. Anderson-Teixeira

# S2 Appendix. TreeHugger failures

TreeHuggers (TH) built in 2015 were installed at the Smithsonian Ecological Research Center (SERC), Wind River Forest Dynamics Plot (WFDP), and Barro Colorado Island (BCI), where we detected six types of failures (detailed below; failure rates given in Table S1 below). TreeHugger technology has changed since these units were built, so failures observed here should not be viewed as indicative of the performance of the current versions. We encourage the reader to contact the vendor (Global Change Solutions, LLC) for the latest information about TreeHuggers.

1. SD card failure. TreeHuggers record data on a micro SD card. These cards need to be removed during data collection and re-inserted into the TreeHuggers’ card holder/reader. What we called a SD card failure was the inability of TreeHuggers to write records on the SD card or the inability of computers to read the SD card. Numbers reported in Table S1 are for failure that happened before or within study period. More SD cards subsequently failed, especially at BCI, probably because of corrosion caused by high humidity.
2. Reset of timestamp. Timestamps for some records suddenly changed to January 2000. This was corrected by hand assuming that the time was still taken at 15-minute intervals.
3. Duplication of timestamps. Some consecutive records were attributed the same timestamp while other measurements showed obvious signs of progression through time (temperature increasing or decreasing). If there were more than four occurrences of duplicated timestamps we deleted all following records.
4. Missing timestamps. No data points were recorded for some timestamps. We did not apply any correction for this.
5. Erratic temperature records. The temperature records from the same time of a day were compared to identify outliers. An outlier was defined as the data point that is located outside 1.5 times the interquartile range above the upper quartile and below the lower quartile. It is worth noting that in some TreeHuggers the outliers occurred at a particular time interval, e.g., 12:00 to 15:00) throughout the study period, perhaps indicating periods of direct sunlight. Outliers were removed from temperature time series, but associated circumference records were not deleted.
6. Unexplained erratic data. For two trees, even after screening of outliers the remaining “clean” time series didn’t reflect plausible tree stem fluctuation patterns. As we suspected electronic deficiency, these trees were removed from analysis.

TreeHuggers built in 2014 and installed at SCBI received a lot of maintenance; they were visited at least one visit a week and problems were fixed as quickly as possible. Because these units are an older version and were managed differently, we do not include them in the statistics presented in Table S1. The following information, collected from a local report (Herrmann et al., unpublished), applies to the period of May to October 2014. TH bands were deployed on trees for a total of 41,705 hours, 40,085 of which yielded in good data (96%). Primary causes of bad data — in order of total data loss — were (1) spikes (visually detected), where data for an entire day were considered unreliable if a spike occurred that day (58% of removed records, nine instances); (2) faulty installation such that the stylus was not in constant contact with the membrane potentiometer (16% of the removed records, one instance); (3) band settling (13% of the removed records, one instance); (4) bear damage (8% of the removed records, three instances); (5) other or unknown problems (*e.g.,* batteries not changed in time, unexplained failure of logger, logger not replaced after problem, loose connection after manipulation or potential bear intrusion; 5% of the removed records). The data records started later for four trees because of logger malfunction or improper installation.

# S2 Table. Failure occurrences for TreeHugger units built in 2015. Units were installed at BCI, SERC, and WRDP.

| Failure type | | SERC | WFDP | BCI | Total |
| --- | --- | --- | --- | --- | --- |
| SD card Failure | Total (leading to complete lack of data) | 9 (5) | 5 (2) | 4 (1) | 18 |
| TimeStamp issue | Reset | 1 | 7 | 1 | 9 |
|  | Duplicated | 1 | 0 | 1 | 2 |
|  | Missing | 2 | 3 | 0 | 5 |
| Temperature record | More than 5% outliers | 7 | 5 | 4 | 16 |
| Manually removed | Data manually deleted after screening | 0 | 1 | 1 | 2 |
| Total |  | **20** | **21** | **11** | **52** |
