# Supplementary material for: Tree Circumference Dynamics in Four Forests Characterized Using Automated Dendrometer Bands
Source: PLoS One. 2016 Dec 28;11(12):e0169020. doi: 10.1371/journal.pone.0169020 (PMC5193451; doi:10.1371/journal.pone.0169020)
Supplement: S3 Appendix — (DOCX) [file pone.0169020.s003.docx]

**Tree circumference dynamics over short time scales using automated dendrometer bands in four forests**

Valentine Herrmann, Sean M. McMahon, Matteo Detto, James A. Lutz, Stuart J. Davies, Chia-Hao Chang-Yang, Kristina J. Anderson-Teixeira

# S3 Appendix. Relationship between *T_band_* and *T_air_*.

The ensemble temperature records (*T_band_*) of each site aligned as expected with air temperature (*T_air_*) measured at a nearby weather station. Based on linear regression analysis, *T_band_* and *T_ai_*_r_ were closely correlated at all sites (all R^2^ ≥ 0.74, Table S2 below). *T_band_* had a slightly smaller daily amplitude (i.e., lower maximums and higher minimums), as is to be expected for understory environments relative to above-canopy conditions or open fields (where T_air_ was measured).

# S3 Table. Comparison of *T_band_* and *T_air_*. Reported are mean temperatures, differences in daily amplitudes (amplitude of *T_air_ -* amplitude of *T_band_*) and linear regression results (df_n_ and df_d_ are numerator and denominator degrees of freedom of the F statistic).

|  | mean *T_band_* | mean *T_air_* | mean difference in daily amplitude | F |  |  | R^2^ | p | slope | | |  | intercept | | |
| --- | --- | --- | --- | --- | --- | --- | --- | --- | --- | --- | --- | --- | --- | --- | --- |
| Site |  |  |  |  | df_n_ | df_d_ |  |  | est | *t* | *p* |  | est | *t* | *p* |
| SCBI | 20.67 | 21.35 | -1.87* | 1.82 10^5^ | 1 | 5943 | 0.97 | <0.001 | 0.78 | 428.2 | <0.001 |  | 3.98 | 100.6 | <0.001 |
| SERC | 23.32 | 24.17 | -2.60* | 1.72 10^4^ | 1 | 5915 | 0.74 | <0.001 | 0.67 | 131.02 | <0.001 |  | 7.20 | 57.94 | <0.001 |
| WFDP | 15.30 | 15.23 | -1.88* | 5.53 10^4^ | 1 | 5950 | 0.90 | <0.001 | 0.84 | 235.13 | <0.001 |  | 2.94 | 51.56 | <0.001 |
| BCI | 25.91 | 25.75 | -1.03* | 5.33 10^4^ | 1 | 6046 | 0.90 | <0.001 | 0.83 | 230.8 | <0.001 |  | 4.41 | 47.3 | <0.001 |

*significantly different, based on paired t-test.
