# Supplementary material for: Tree Circumference Dynamics in Four Forests Characterized Using Automated Dendrometer Bands
Source: PLoS One. 2016 Dec 28;11(12):e0169020. doi: 10.1371/journal.pone.0169020 (PMC5193451; doi:10.1371/journal.pone.0169020)
Supplement: S4 Appendix — (DOCX) [file pone.0169020.s004.docx]

**Tree circumference dynamics over short time scales using automated dendrometer bands in four forests**

Valentine Herrmann, Sean M. McMahon, Matteo Detto, James A. Lutz, Stuart J. Davies, Chia-Hao Chang-Yang, Kristina J. Anderson-Teixeira

# S4 Appendix. Thermal expansion experiment

Metallic dendrometer bands are subject to lengthening due to thermal expansion, which needs to be quantified to estimate true diameter change [1,2]. However, a simple correction for thermal expansion of the stainless steel band—*i.e.,* applying a linear expansion correction factor based on *T_air_*—can be inappropriate considering the physical complexity of the system. Major factors that may make a simple correction inappropriate include thermal expansion of the tree [2], friction between the band and the tree, and thermal inertia of the tree and band (so *T_band_* ≠ *T_air_*). A simple correction that fails to account for these factors could overcorrect for temperature. To determine whether the TreeHuggers could show a signal of daily shrinkage attributable to thermal expansion of the steel banding, we conducted a simple experiment to observe the temperature response of bands tensioned around a cylindrical object with a lower thermal expansion coefficient, less friction, and lower thermal inertia than a tree. Specifically, we installed ADBs on two cylindrical glass vases with diameters of 20 cm and 40 cm in an open garage on the SCBI campus, protected from sun flecks but subject to thermal variation. This experiment was run from July 01-15, 2015. Since glass vases had no consistent pattern in ∆*c_r_* in relation to *T_band_* (see Table 3 in main paper), we concluded that it would not be appropriate to apply a thermal correction to the dendrometer band readings.

1. Wang J, Sammis TW. New automatic band and point dendrometers for measuring stem diameter growth. 2008; 1–20.

2. Sevanto S, Hölttä T, Hirsikko A, Vesala T, Nikinmaa E. Determination of thermal expansion of green wood and the accuracy of tree stem diameter variation measurements. Boreal Environ Res. 2005;10: 437–445.
